# Molting site fidelity accounts for colony elimination of the Formosan subterranean termites (Isoptera: Rhinotermitidae) by chitin synthesis inhibitor baits

**DOI:** 10.1038/s41598-018-19603-8

**Published:** 2018-01-19

**Authors:** G. Kakkar, W. Osbrink, N.-Y. Su

**Affiliations:** 1 University of Florida-IFAS Extension, St. Lucie and Indian River County, 8400, Picos Road Ste. 101, Ft. Pierce, FL 34945 USA; 2 U.S. Livestock Insects Research Laboratory, USDA-ARS-SPA Knipling-Bushland, 2700 Fredericksburg Rd., Kerrville, TX-78028 USA; 3 Department of Entomology and Nematology, Institute of Food and Agricultural Science, Fort Lauderdale Research and Education Center, University of Florida, Davie, FL-33314 USA

## Abstract

Site fidelity by molting termites in Formosan subterranean termite, *Coptotermes formosanus* Shiraki colonies is a new addition to our understanding of lower termites’ behavior and biology. Our previous studies indicated that workers moved to the central nest to molt in the presence of eggs and reproductives. The current study showed that noviflumuron-affected workers also return to the central nest and died in the vicinity of reproductives and eggs. The aversion to the dead and decaying workers caused reproductives and brood to leave the original central nest site in a colony and refuge at newer sites every few days in response to newly dead workers near them. Because mortality was an event observed only in workers undergoing molting under the effect of noviflumuron- a CSI, the death of molting individuals was observed only around reproductives and brood. This study reveals a previously undiscovered behavior of molting termites and the mechanics behind a successful arsenal; noviflumuron baits used against subterranean termites.

## Introduction

There are over 3,106 described species in the order Isoptera, and ~6% of them are known as economically important pests^1^. *Coptotermes formosanus* Shiraki is one of the important representatives from the list, and it has become a serious pest because of its large colony size and worldwide distribution. It is an adventive pest in the United States, and the colony mainly lives in soil and nest underground or in trees where the colony is composed of the main nest and/or satellite nests interconnected by a gallery system^2,3^. Inside these galleries, termites forage for food and enter structures from the surrounding soil. Because of the large population size and cryptic nature of subterranean termites, it is hard to detect the invasion until there are external signs of infestation above ground.

Amongst strategies available for control of subterranean termites, chitin synthesis inhibitor (CSI) incorporated baits have been demonstrated to be a successful method for elimination of subterranean termite colonies^4^. To eliminate a subterranean termite colony, a bait has to be ingested by foraging termites and distributed throughout the colony population, and it was suggested early on that an active ingredient (AI) has to be non-repellent and slow-acting to avoid the accumulation of dead bodies near a bait station^5^. Many AIs have been reported as potential bait toxicants, but field trials with slow-acting and non-repellent metabolic inhibitors such as diiodomethane para-tolyl sulfone, sulfluramid, and hydramethylnon did not eliminate colonies of subterranean termites^6–8^. Results of these field trials prompted Su *et al*.^7^ to suggest that failures by the metabolic inhibitors to eliminate target colonies was attributable to their dose-dependent lethal time. AI concentrations in bait matrix can be adjusted, but once a bait is placed in the field, the amount of bait consumed by individual termites may vary among foragers. Because the lethal time of a metabolic inhibitor is dose-dependent, those consumed a large quantity of bait may be killed quickly, negating the slow-acting requirement of a successful bait AI. Hence, in addition to non-repellence and slow-action, lethal time of a bait AI needs to be dose-independent if the objective is to eliminate a termite colony^9^.

CSIs like noviflumuron interfere with the formation of cuticle and affect the molting process of workers, but those ingested lethal doses of CSIs are not affected until the onset of ecdysis, independent of dose. This may explain, in part, why only baits incorporating CSIs were successful in eliminating termite colonies as reported in many field studies^4,10^. A recent study by Kakkar *et al*.^11^ showed that termites move back to central nest near reproductives and brood to complete the molting process and this molting-site fidelity may offer another explanation for the success of noviflumuron baits to eliminate termite colonies. Because molting took place in the central nest, we hypothesize that noviflumuron-affected termites may die near reproductives and broods instead of the foraging sites, which prevents the aversion of dead termites at bait stations. This study was initiated to test this hypothesis.

## Results and Discussion

**Figure 1:**
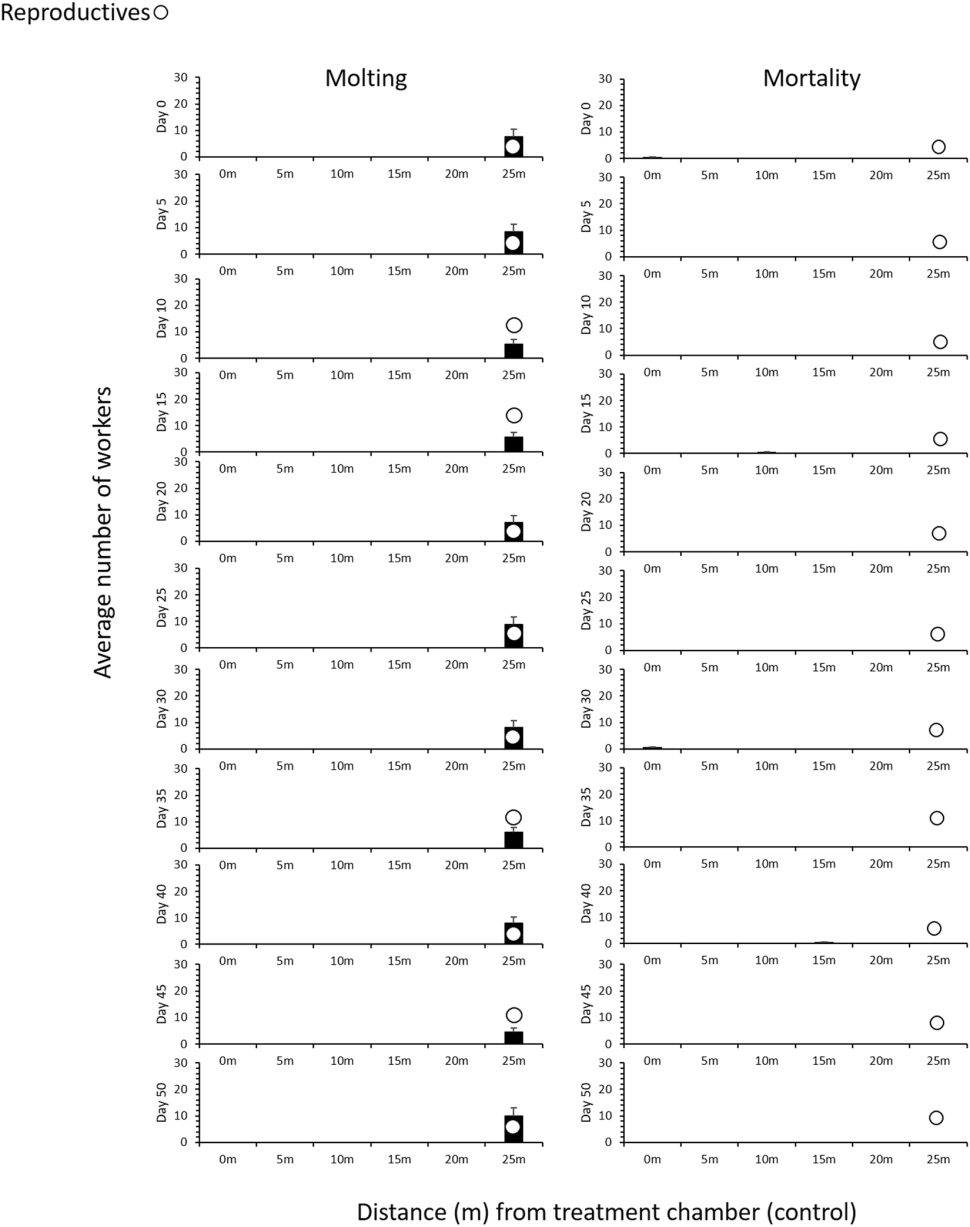
Mean number (±S.E) of worker molting (left) and worker mortality (right) in three 6 yr old juvenile colonies (**A**,**B**, and **C**) using 25 m long linear foraging arena for control.

Of the two main effects (treatment and time), only treatment had a significant effect on the count of molting and mortality. No significant interaction of treatment × time was observed. On average 79 ± 2.6 (Mean ± SE) workers successfully molted in a control colony throughout the study period (Table 1). Only white workers molted during the initial 10 days, indicating that these were under the pre-ecdysis fasting when the feeding pads stained with Nile Blue A were placed in the 0 m chamber^12,13^. After 10 d, blue workers that fed on dyed feeding pads started to molt. Molting occurred exclusively in the central nest in all control colonies, which corroborates with the molt-site fidelity in *C. formosanus* colonies described by Kakkar *et al*.^11^. Workers traveled from the foraging site to the central nest for molting and stayed inside or in the vicinity until at least 36 h post molting. In addition to the 1.3 ± 0.6 (Mean ± SE) dead workers in control colonies (Table 1), no freshly dead workers were recorded in any of the control arenas throughout the test period (Fig. 1). We suspect that dead termites were either buried or cannibalized by the nestmates between the 5-d intervals as reported in other studies^12,14–16^.

**Table 1:** Mean (±SE) number of workers molting and workers dead in control and noviflumuron treated colonies during the study period.

Treatment	Workers successfully molting	Workers dead
Control	79 ± 2.6	1.3 ± 0.6
Noviflumuron	18 ± 3.7	147 ± 13
Statistical analysis	F = 20.31; P < 0.0001	F = 26.29; P < 0.0001

**Figure 2:**
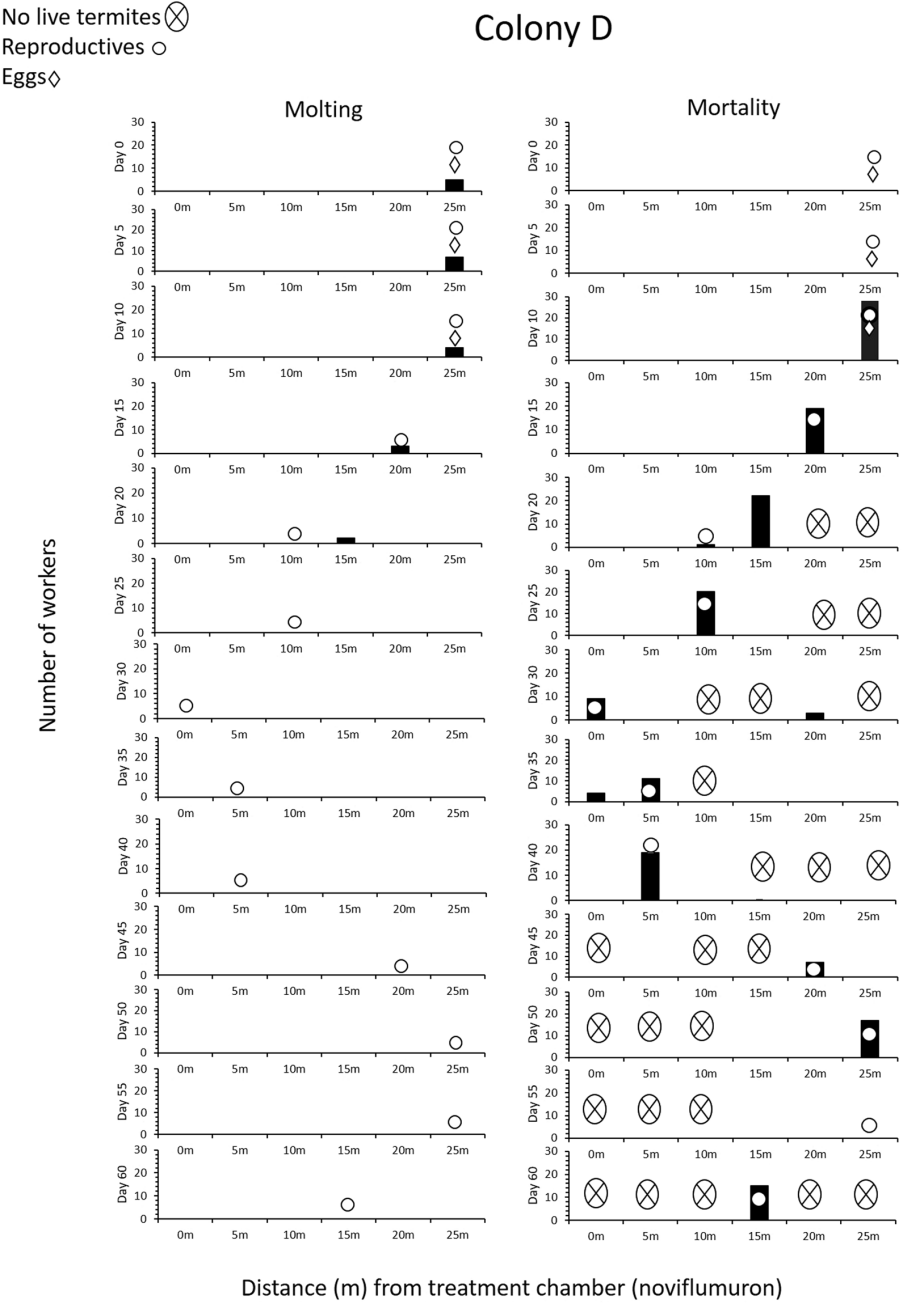
Number of worker molting (left) and worker mortality (right) in a 6 yr old juvenile colony (D) for noviflumuron treatment.

**Figure 3:**
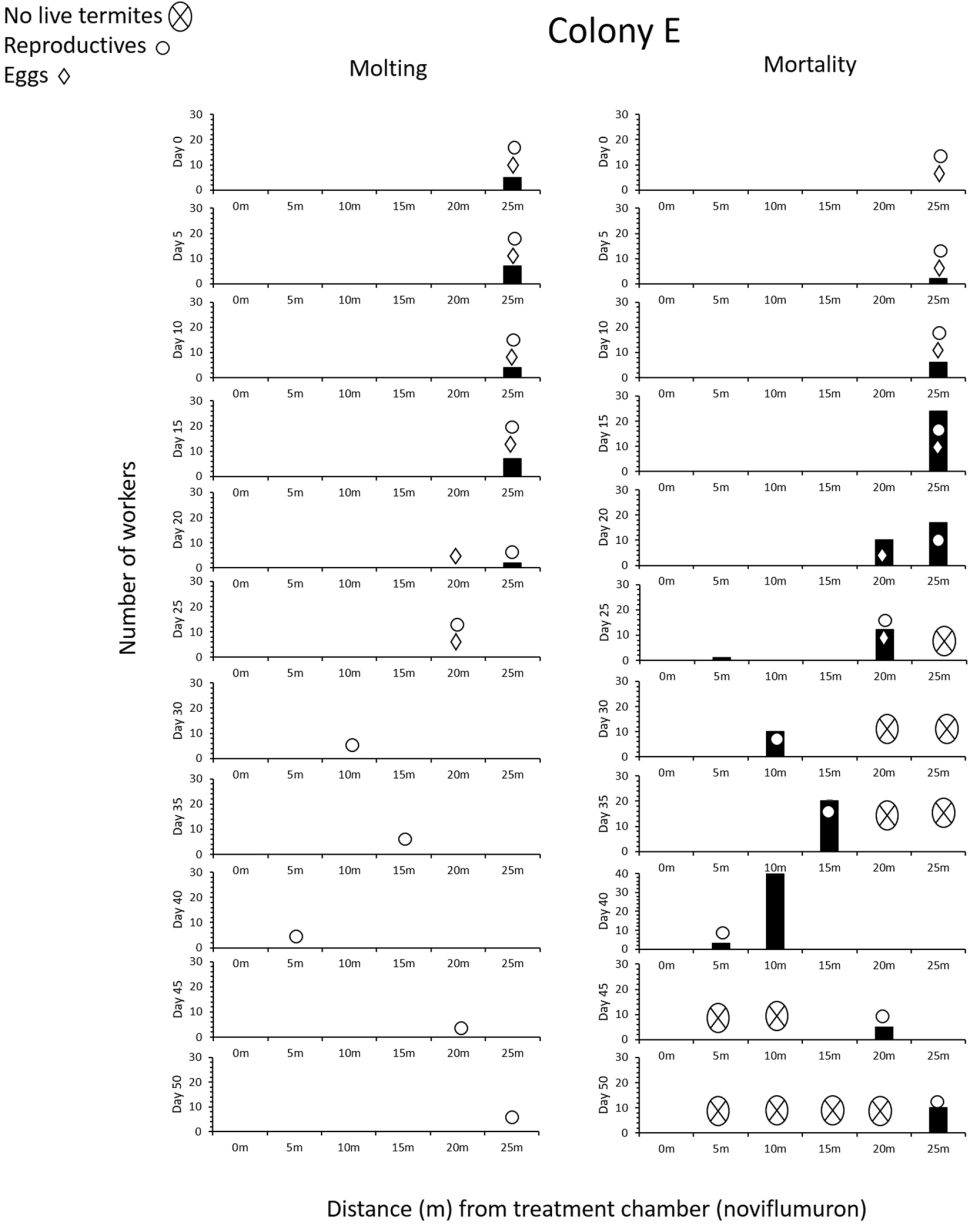
Number of worker molting (left) and worker mortality (right) in a 6 yr old juvenile colony (E) for noviflumuron treatment.

**Figure 4:**
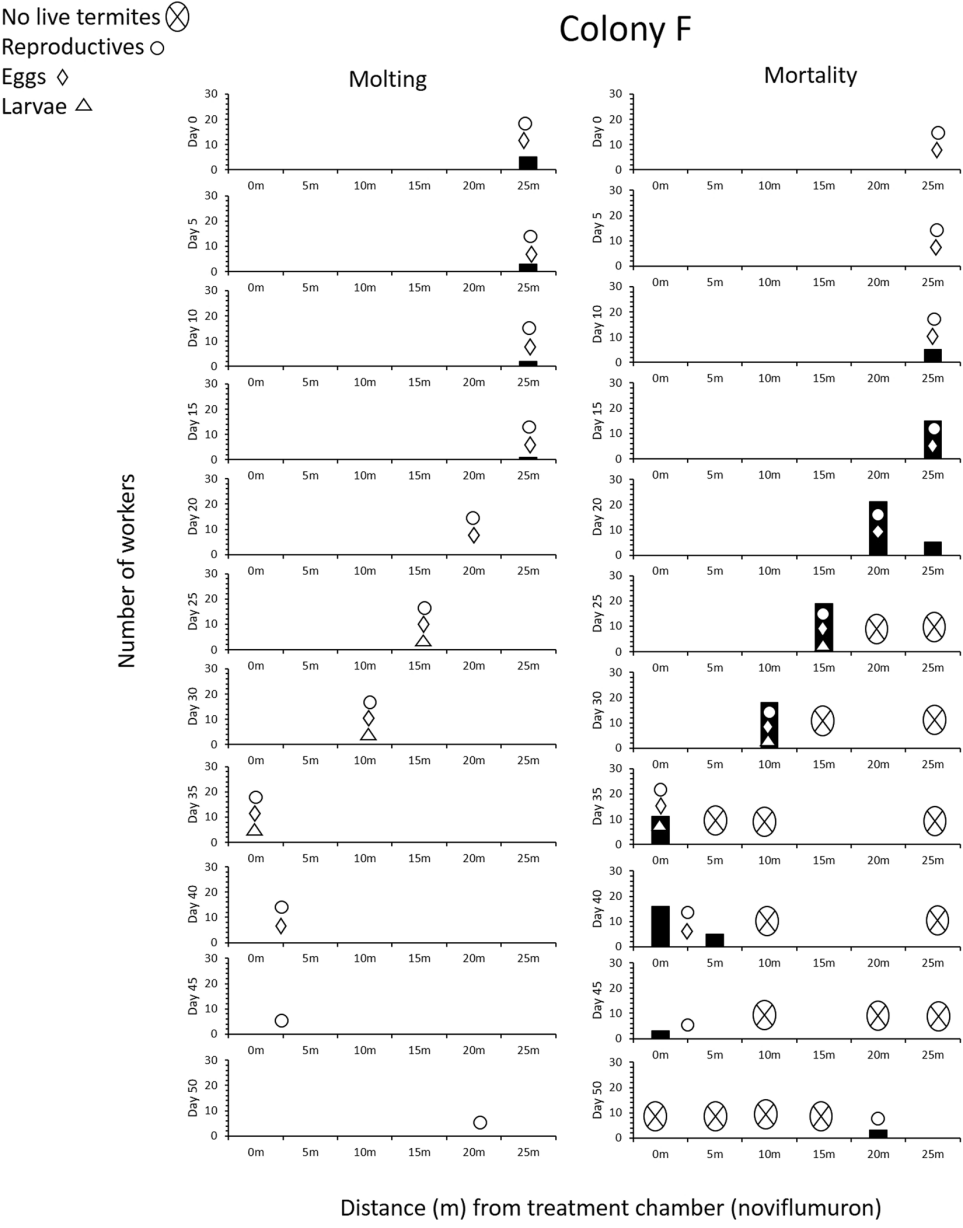
Number of worker molting (left) and worker mortality (right) in a 6 yr old juvenile colony (F) for noviflumuron treatment.

Unlike control group, successful molting in noviflumuron treated colonies was observed only for initial 20 d, 20 d, and 15 d for colony D, E, and F, respectively (Figs 2–4). Only white workers, which did not feed on noviflumuron due to their pre-ecdysis fasting were able to molt successfully. However, over time there was a decline in the number of successfully molted workers in noviflumuron treated colonies because of the spread of noviflumuron to the entire colony as implied by the increase of blue workers. The number of successful molts was significantly lower than workers molting in control colonies (*P* < 0.05) (Table 1). In colony D, reproductives and eggs were found in the 25 m arena for the initial 10 d, and molting termites were also found in the same arena (Fig. 2). The three treated colonies collapsed within 60 d after the addition of noviflumuron baits to the arenas, as dictated by the age-dependent molt-cycle of the oldest workers in the colonies^17^.

**Figure 5:**
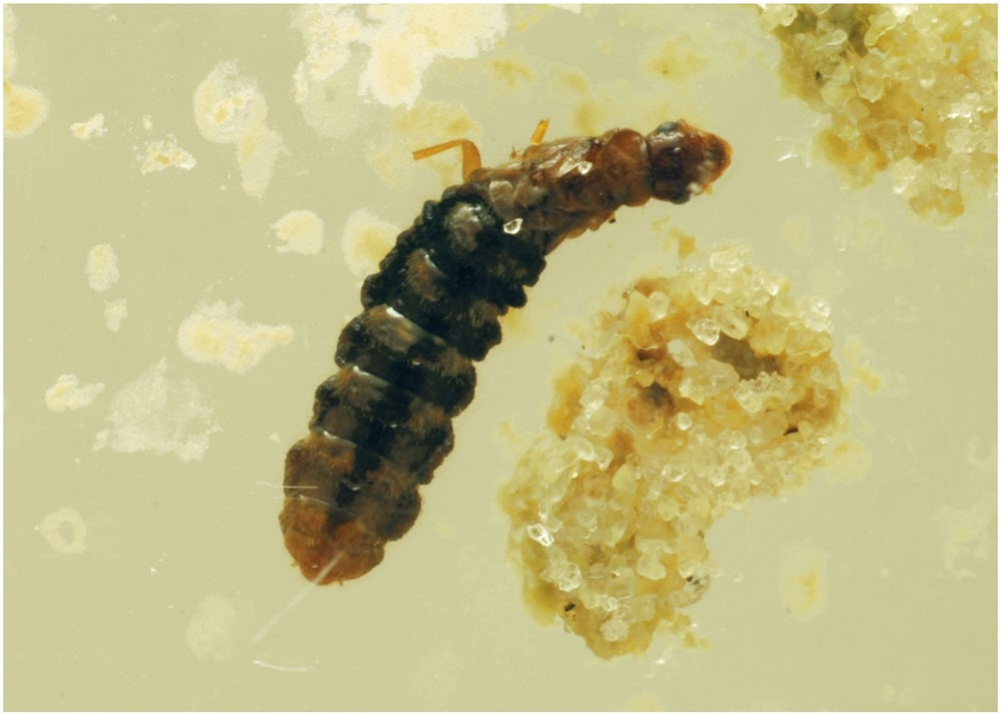
Dead queen with lost legs due to excessive grooming by workers in colony ‘D’ treated with noviflumuron.

In colony D, as workers started to die in an attempt to molt on day 10 in the 25-m arena, eggs were moved by workers on several occasions within the same 25-m arena. Molting workers of *C. formosanus* are known to have an affinity towards the eggs^11^, and we suggest that accumulation of corpses around eggs in the 25-m arena (Fig. 2) may have led to aversion, resulting in the transportation of eggs by workers, a behavior commonly seen in other termite species as well^18,19^. The presence of corpses and possibly decomposition volatiles such as indole or phenol^20^ promoted reproductives to move from 25-m arena to 20-m arena by day 15 (Fig. 2), during which eggs had disappeared and were probably cannibalized. Cannibalism was probably to bring stability while dealing with a rapid loss of workers and that the taskforce dedicated to nurturing the brood could be used to attend and nourish the reproductives. Following the reproductives, molting termites also moved to 20-m arena on day 15 (Fig. 2). Due to the effects of noviflumuron, the molting termites in 20-m died, and between day 15 and day 20, the reproductives probably first moved to the 15-m arena and then to the 10-m arena by day 20 when dead termites accumulated in the 15-m arena. A small number of molting termites also followed the movement of reproductives first to the 15-m arena, but due to ecdysis they probably could not reach the 10-m arena, and instead were found in the 15-m arena (Fig. 2). By day 20, no live termites were found in the 20-m and 25-m arenas and cadavers were covered with fungus. After day 20, no molting termites were found in any of the arenas, but reproductives were always surrounded by corpses and continued to move, i.e., from 0-m arena (day 30) to 5-m arena (day 35 and 40), back to 20-m (day 45) and 25-m area (day 50 and 55), and eventually died (Fig. 5) in the 15-m arena by day 60. By day 60, all the workers were dead, but 97 live soldiers were seen in the treatment chamber and 15-m arena, which also died eventually.

Similar patterns of reproductives moving from arena to arena, presumably to avoid dead termites were also observed in colonies E and F (Figs 3, 4). Reproductives, eggs and molting termites of colony E were all found in the 25-m arena until day 15 when a large number of dead termites appeared there (Fig. 3). Eggs were transported to the 20-m arena by day 20, and reproductives also moved to the same arena by day 25, but both were eventually surrounded by corpses. Between day 25 and 30, eggs were apparently cannibalized, and reproductives were found to reside in different arenas each time the arenas were surveyed, i.e., 15-m, 5-m, 20-m arenas on day 35, 40, and 45, until male and female reproductive died in the 25-m arena on day 45 and 50, respectively. By day 50, no live workers were found in the arenas and colony had crashed.

**Figure 6:**
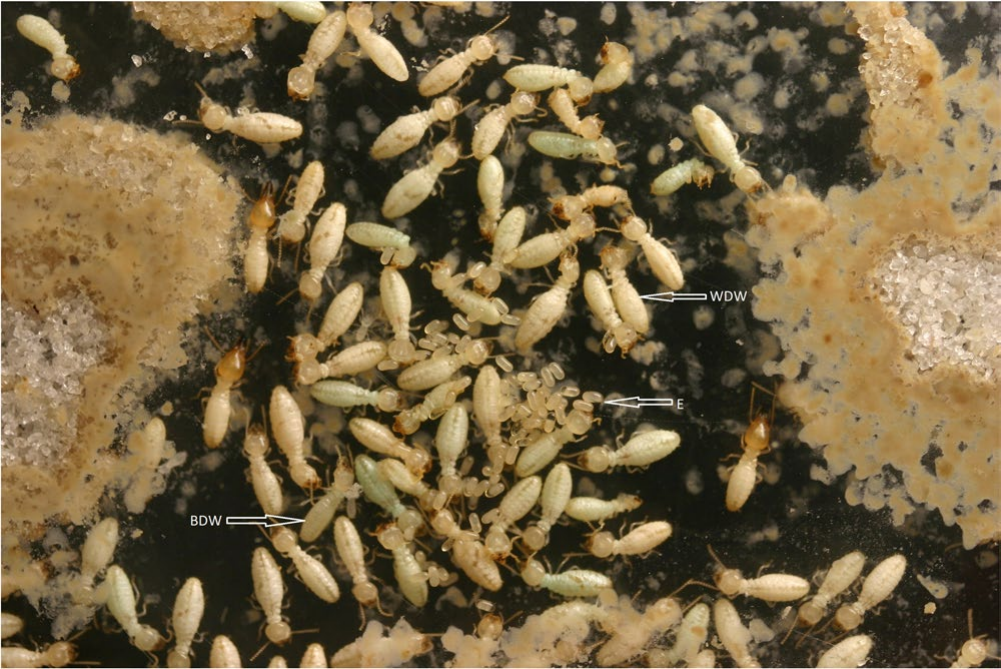
Planar arena containing eggs, soldiers (live), and workers (live and dead) from a colony treated with noviflumuron. ‘E’ signifies eggs, ‘WDW’ signifies white dead worker, and ‘BDW’ signifies blue dead workers.

For colony F, molting termites were found in the 25-m arena near the reproductives and eggs until day 15 (Fig. 4). As with the colonies D and E, reproductives and eggs moved to the other arenas when surrounded by corpses. Despite worker mortality, some eggs hatched and larvae were found in the 15-m arena along with reproductive by day 25 (Fig. 6). Larvae were transported with eggs to the same arenas as the reproductives, but eventually, no larvae were found by day 40, and eggs also disappeared by day 45. Toward the final day, eggs and reproductives were found in the Tygon tubing between 0-m and 5-m arena until day 40 and 45. Reproductives stayed inside the tubing until there was an accumulation of dead workers around them. Eventually the reproductives moved to the 20-m arena which was earlier abandoned by them and died there (Fig. 4). By day 50, only 88 live soldiers were found in the 20 and 25 m arena. In all three treated colonies, soldier (80 ± 12.7; Mean ± S.E) was the only live caste observed towards the end.

The number of dead workers observed in treated colonies D, E, and F was significantly higher than workers died in control colonies A, B, and C (*P* < 0.05) (Table 1). In the third week after adding noviflumuron to colonies, workers developed signs of noviflumuron intoxication recognized as white subcuticular accumulations of uric acid throughout the fat body, referred as marbling^14,15,21,22^, unlike control colonies where workers were healthy throughout the study period.

To summarize, results show that noviflumuron affected workers moved to central nest and died in the attempt to molt, promoting reproductives to move to other locations only to be surrounded again by another group of dead or dying workers during their ecdysis. The reproductives were chased by dead termites and at the same time distributed the corpses at various locations in the gallery system as they moved. The dispersion of physogastric queens is typical in other termite species as well. Queens are reported to relocate as a defense mechanism when threatened by predators^18^ or when a colony (*Nasutitermes sp.)* decides to abandon the old nest and transfer to a new nest^19^. This implies that in a field colony of *C. formosanus* that received noviflumuron baits unless the central nest resides close to the bait station, mortality and accumulation of cadavers will always be away from the bait station resulting in the absence of bait aversion to ensure the continuing spread of lethal dose of noviflumuron throughout a colony.

Spatial progression of death and response of nestmates including reproductives, eggs, and larvae to mortality in a colony baited with noviflumuron has an important implication in speeding up the elimination of baited colonies of *C. formosanus*. In the past, the slow process of colony elimination with the use of CSI baits has raised concerns^10,13,23^, and several attempts have been made to improve the bait efficacy. One attempt at enhancing the effect of CSI in baits was the combination of ecdysteroids or molt accelerating compounds (MAC) with noviflumuron in baits^23,24^. The two compounds, when tested on subterranean termites in the laboratory, were found to have an enhanced effect compared with the noviflumuron or MAC alone. These resulted in accelerated/premature molting in the workers under the effect of MAC, which at the time of molting had poorly formed new cuticle under the effect of noviflumuron, resulting in failed molting leading to death.

Based on our results confirming that molting site fidelity continues in colonies treated with noviflumuron, we suggest that mortality due to noviflumuron will always initiate in the central nest of a colony instead of a foraging site or the baiting site and there will be no secondary repellency at the bait station which will allow for noviflumuron lethal dose to spread in a colony resulting in its elimination. Also, the ability of workers with an acquired lethal dose of noviflumuron reaching the nest before molting incidence implies that upon adding molt inducing chemicals to induce early molting, regardless of how fast it is, there is a scope to reduce the lethal time for colony elimination without any bait station aversion. In conclusion, our findings on termite molt behavior will ensure that the efficacy of noviflumuron baits that have been accelerated with the use of MAC will not cause secondary station aversion and will achieve similar colony elimination.

## Materials and Methods

Six, 7-yr old (juvenile) colonies of *C. formosanus* were used in this study. Colonies were initiated using alates collected from the swarming events in New Orleans in 2007. The alates were paired and placed in 10-dram polystyrene vials (8 × 9 × 2.5 cm^3^) containing pieces of wood (*Picea* sp., 0.6 × 0.6 × 5 cm^3^) and moistened soil, and kept at 29 ± 1 °C. Over the time, colonies grew, and these vials were transferred to a plastic box (17 × 12 × 7 cm^3^) stored at 29 ± 1 °C.

**Figure 7:**
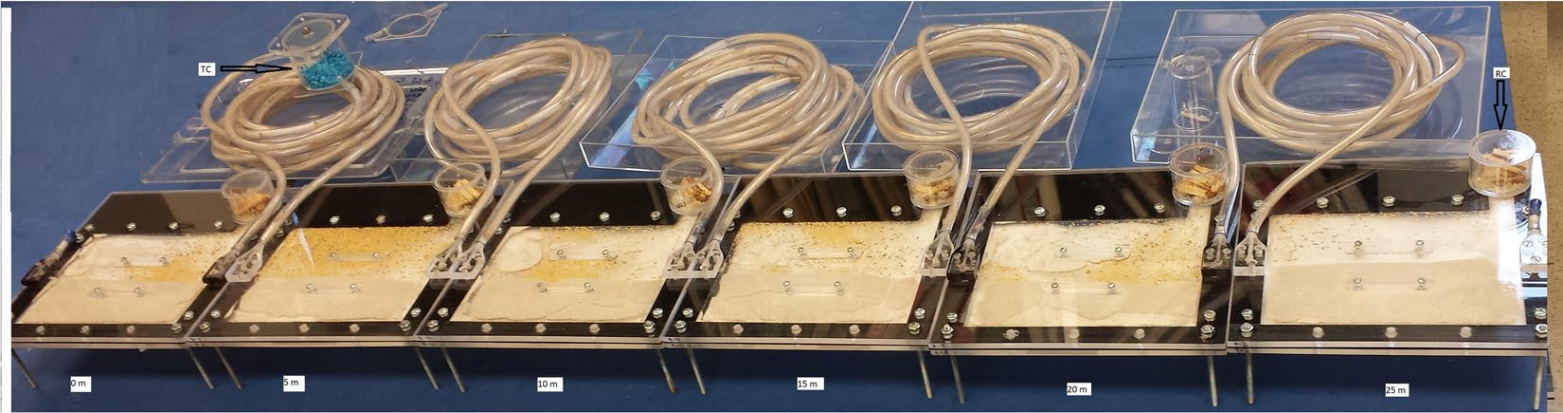
The extended foraging arena was composed of six planar arenas (24 × 24 × 1.4 cm in thickness) filled with moistened sand and connected to each other by a 5 m long coiled Tygon tubing to form a linear distance of 25 m. In the figure, ‘RC’ signifies release chamber and ‘TC’ signifies treatment chamber.

The planar arena was made of two clear sheets of Plexiglass and a spacer (Plexiglas laminate of 0.2 cm thickness) to maintain the 0.2 cm thick inner space for movement of termites between sheets. It was filled with ≈80 g of oven dried sand that was then moistened before use (65 g sand + 15 ml of sterile deionized water). Extended foraging arena similar to that of Su^14^ was composed of six planar arenas connected to each other by 5-m long (12 pieces of 0.5 m long tubes) coiled Tygon tubing (0.6 cm in diameter) to form a linear foraging distance of 25 m (Fig. 7). Termites were introduced to the planar arena through the opening on top sheet that served as a release chamber. The plastic box (rearing unit) containing the juvenile colony was placed on top of the release chamber and a moist piece of wood (*Picea* spp.) was positioned so that one end reached the opening in the release chamber and the other end was joint to the planar arena. The wood served as a substrate for termites to travel from box to the planar arena. As colony started moving into the planar arena, the setup was extended in one direction, and five additional planar arenas of the same size as the first were connected through 5 m long Tygon tubes, forming a 25 m long linear foraging arena. Primary reproductives and eggs moved and stayed inside the original planar arena, and it was referred as the central nest. The plastic box was removed once the colony members moved into the foraging setup and the release chamber was filled with moistened wood pieces to provide food sources before closing it with a perforated plastic lid to allow aeration and avoid any termites escaping from the setup.

The foraging arena was maintained in the dark at 27 ± 0.5 °C and termites were allowed to settle in the arena. After 2 wk, a treatment chamber (6 cm in diameter and 8 cm in height) was added to the corner most planar arena placed on the other end of the foraging arena containing reproductives and eggs, the central nest (Fig. 7). The treatment chamber was added to the Tygon tubes connecting the corner most planar arena to the adjacent planar arena. For each treatment (control and noviflumuron), three juveniles colonies were used as the replicates, totaling six colonies for the study. For control, colonies (A, B, and C) were provisioned with cellulose pads dyed with Nile Blue A (0.05% wt/wt), and for treatment, colonies (D, E, and F) were treated with cellulose pellets containing 0.5% noviflumuron and dyed with Nile Blue A (0.05% wt: wt). The dye was added to confirm termite feeding from the treatment chamber.

The planar arena closest to the treatment chamber was referred to as 0 m arena, and others as 5 m, 10 m, 15 m, 20 m, 25 m in reference to the treatment chamber, hence the central nest was initially located at 25 m from the treatment chamber. Visual count of workers (blue and white) in the process of molting and the ones which have recently molted based on their mandible color due to varying levels of sclerotization^25^ were made in both control and treatment units. Also, visual counts of any freshly dead workers, moribund (laid on side or back), and workers in molt-inhibitory postures formed under the effect of noviflumuron^26^ were recorded. Counts were recorded from each planar arena across 25 m long foraging setup starting from the day treatment chamber (cellulose pads/noviflumuron + 0.05% Nile Blue A) was added to the unit at five days interval until the colony collapsed (0 d, 5 d, 10 d, and so on). Because termites may feed on or bury corpses, counts included only freshly dead workers in each planar arena observed at the time of data collection.

### Data analyses

Comparisons for the molting and mortality count (=variable) between control and noviflumuron treated colonies on all sampling dates for each colony were analyzed independently using a repeated measure one-way ANOVA in a generalized linear mixed model with the SAS^27^ procedure GLIMMIX. The model was used to determine the effect of treatments, sampling period (time), and their interaction on molting and mortality amongst workers, separately. Because the response variable was count data with no upper bound, in the model statement distribution was specified as Poisson. Data were transformed using log_10_ (x + 1) to homogenized variance before analysis. Each termite colony represented a replicate for a total of three replicates. Data presented in table and figures represents un-transformed means of molting and mortality.
